# Progressive Systemic Sclerosis-Associated Interstitial Lung Disease With Clinical-Radiographic Dissociation and Delayed Transplant Access: A Case Report

**DOI:** 10.7759/cureus.86412

**Published:** 2025-06-20

**Authors:** Nicolas Bakinde, Antoinette Johnson, Deborah Ngo Bakinde, Priscilla Pemu, Eric Flenaugh

**Affiliations:** 1 Department of Medicine, Morehouse School of Medicine, Atlanta, USA; 2 General Internal Medicine, Grady Memorial Hospital, Atlanta, USA; 3 Biological Sciences, Savannah State University, Savannah, USA; 4 Pulmonary and Critical Care Medicine, Grady Memorial Hospital, Atlanta, USA

**Keywords:** access to care, clinical-radiographic dissociation, lung transplantation, non-specific interstitial lung disease (nsip), systemic sclerosis (ssc)

## Abstract

Systemic sclerosis-associated interstitial lung disease (ILD) is a major contributor to morbidity and mortality in patients with diffuse systemic sclerosis. We present the case of a 47-year-old man with fibrotic non-specific interstitial pneumonia who experienced worsening hypoxemia and dyspnea following COVID-19, despite ongoing treatment with mycophenolate mofetil and prednisone. His oxygen requirement increased from 6-8 L/minute to 15 L/minute at rest, even as imaging showed stable fibrosis with improvement in ground-glass opacities, highlighting a clinical-radiographic dissociation. Given concern for progressive fibrosing ILD, immunosuppression was escalated to intravenous cyclophosphamide. Referral for lung transplantation was delayed due to insurance non-acceptance at the region’s transplant center, compounding the urgency of his decline. This case underscores the importance of timely recognition of progressive ILD, the limitations of radiographic assessment alone, and the impact of systemic barriers on access to life-saving interventions.

## Introduction

Systemic sclerosis-associated interstitial lung disease (SSc-ILD), a form of progressive lung scarring caused by autoimmune connective tissue disease, is now recognized as a leading cause of morbidity and mortality in patients with diffuse cutaneous systemic sclerosis. While immunosuppressive therapies such as mycophenolate mofetil are often effective, some patients develop a progressive fibrosing phenotype despite standard treatment [1]. Recognizing early indicators of deterioration, particularly when clinical decline outpaces radiographic findings, is crucial for timely intervention and referral for transplant.

In patients with refractory SSc-ILD, disease progression can be rapid and unpredictable, and lung transplantation may represent the only definitive intervention. However, patients with systemic sclerosis often face multiple barriers to transplant eligibility. These include esophageal dysmotility, pulmonary hypertension, renal dysfunction, and frailty, all of which can delay listing or result in exclusion from consideration. This concern is underscored by findings from a retrospective cohort study at Kyoto University, which reported a 24% waitlist mortality rate among patients with SSc-ILD listed for lung transplantation, with only 34% ultimately undergoing transplant [2]. Even among those who receive a transplant, outcomes remain guarded. A national cohort study reported that patients with SSc-ILD had a 48% higher risk of one-year post-transplant mortality compared to those with non-scleroderma interstitial lung disease (ILD) (hazard ratio = 1.48; 95% confidence interval = 1.01 to 2.17) [3]. These findings reinforce the importance of early transplant referral before irreversible clinical decline occurs.

This case illustrates the complexity of managing refractory SSc-ILD in the post-COVID-19 era and highlights the impact of delayed transplant access due to systemic insurance-related barriers.

## Case presentation

A 47-year-old man with a history of diffuse systemic sclerosis complicated by ILD (non-specific interstitial pneumonia (NSIP) pattern), chronic hypoxemic respiratory failure, and heart failure with recovered ejection fraction presented with worsening dyspnea, increased supplemental oxygen requirements, and productive cough. He had been discharged nine days earlier following an admission for COVID-19 pneumonia, during which he received baricitinib, remdesivir, and dexamethasone. At baseline, he required 6-8 L of oxygen via a nasal cannula at home, but in the days leading up to readmission, he reported needing 13-15 L with minimal exertion.

**Figure 1:**
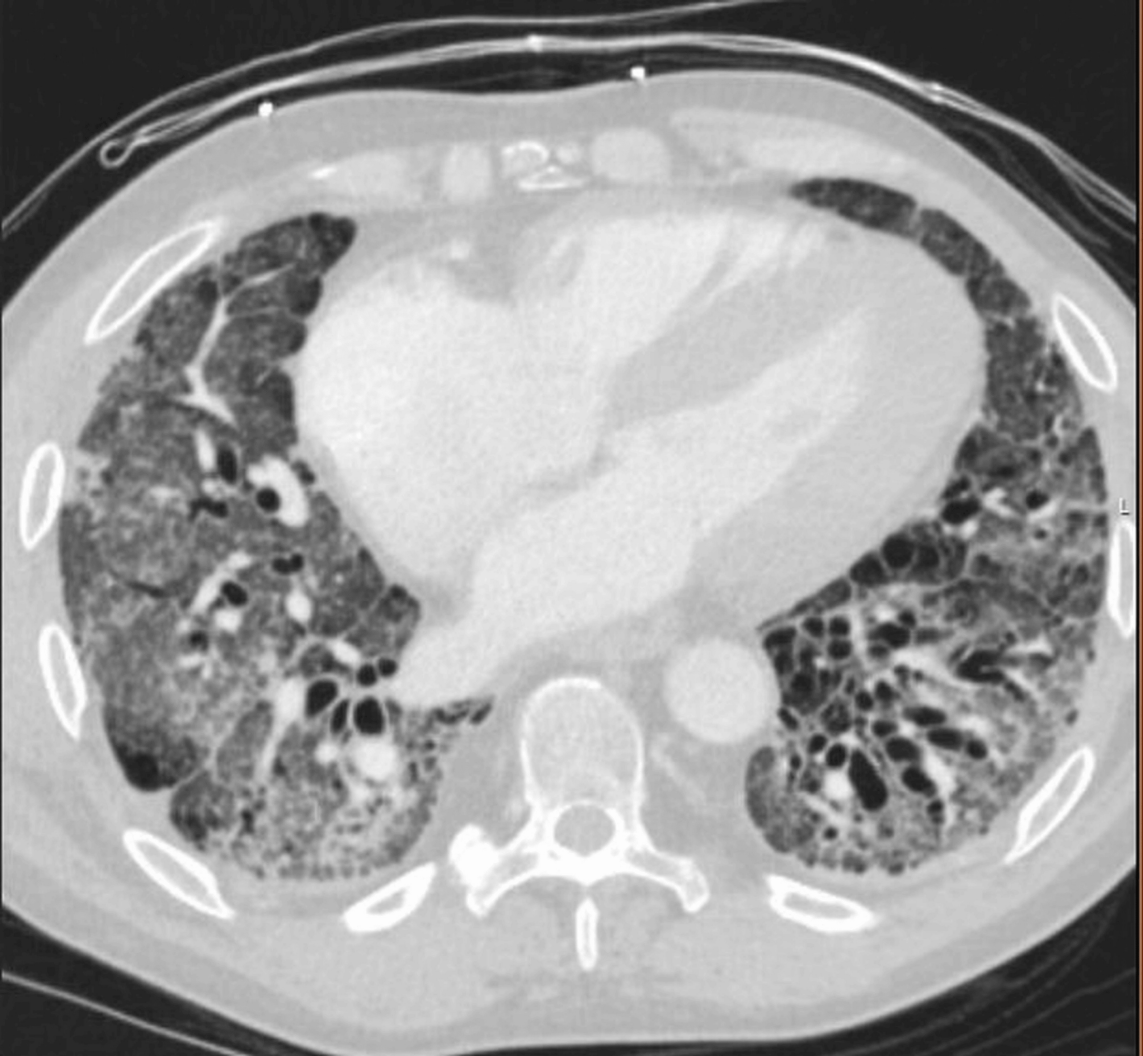
Axial chest CT. High-resolution axial CT image demonstrating bilateral subpleural reticulation, traction bronchiectasis, and fibrotic changes consistent with non-specific interstitial pneumonia. These findings are most prominent in the posterior and basilar regions.

On presentation, he was tachycardic and hypoxemic but afebrile and hemodynamically stable. Physical examination revealed bibasilar crackles, mechanic’s hands, and skin thickening of the face and extremities. CT imaging demonstrated no pulmonary embolism and interval improvement in ground-glass opacities, though there was stable fibrotic NSIP with traction bronchiectasis and dilation of the main pulmonary artery (Figure 1).

Infectious workup, including bacterial cultures, respiratory viral PCR, and fungal biomarkers, was unrevealing aside from a transiently positive SARS-CoV-2 PCR, likely reflecting persistent viral shedding. Laboratory evaluation showed leukocytosis, thrombocytosis, elevated inflammatory markers, and stable renal function.

Despite ongoing treatment with mycophenolate mofetil and prednisone, the patient experienced clinical and functional deterioration. Given concern for progressive fibrotic disease, immunosuppression was escalated to intravenous cyclophosphamide. Lung transplant evaluation was pursued, but the patient’s insurance was not accepted at the region’s primary transplant center, creating a delay in accessing definitive care. His case exemplifies the intersecting clinical and structural barriers that complicate the management of end-stage SSc-ILD.

Diagnostic assessment

In the setting of tachycardia, worsening hypoxemia on high-flow oxygen, and mild respiratory distress, a comprehensive diagnostic assessment was performed to evaluate for infectious, inflammatory, cardiac, or fibrotic progression.

Laboratory evaluation revealed persistent leukocytosis (peak white blood cell count of 19.1 × 10⁹/L) and thrombocytosis (platelet count up to 621 × 10⁹/L), normocytic anemia (hemoglobin nadir of 8.6 g/dL), and elevated inflammatory markers, including C-reactive protein and lactate dehydrogenase (384 U/L). Procalcitonin was low (0.14 ng/mL), and serum lactate was normal. Renal function remained stable (creatinine of 0.8-1.0 mg/dL), and there was no laboratory evidence of scleroderma renal crisis. Cardiac biomarkers showed a mildly elevated brain natriuretic peptide (449 pg/mL) and a stable, low-level troponin elevation without ischemic symptoms. A summary of select laboratory results at key timepoints is provided in Table 1.

**Table 1:** Select laboratory results at key timepoints. This table summarizes key laboratory findings during the index admission for progressive hypoxia and in the immediate prior hospitalization for COVID-19 pneumonia. Values are presented alongside standard reference ranges. Labs reflect systemic inflammation, cardiac strain, and stable renal function. A transiently positive SARS-CoV-2 PCR was attributed to persistent viral shedding. WBC = white blood cell count; CRP = C-reactive protein; LDH = lactate dehydrogenase; BNP = brain natriuretic peptide

Timepoint	Laboratory test	Result	Reference range
On admission	WBC	19.1 × 10⁹/L	4.0–11.0 × 10⁹/L
	Hemoglobin	8.6 g/dL	13.5–17.5 g/dL
	Platelets	621 × 10⁹/L	150–450 × 10⁹/L
	CRP	Elevated	<5 mg/L
	LDH	384 U/L	140–280 U/L
	Troponin	Mildly elevated	<0.04 ng/mL (assay-specific)
	BNP	449 pg/mL	<100 pg/mL
	Creatinine	0.8–1.0 mg/dL	0.7–1.3 mg/dL
	Procalcitonin	0.14 ng/mL	<0.25 ng/mL
During prior admission	SARS-CoV-2 PCR	Transiently positive	

**Figure 2:**
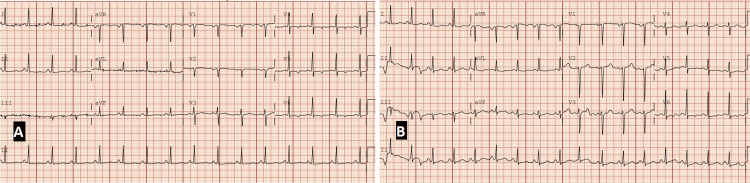
Baseline and admission electrocardiograms demonstrating evolving cardiopulmonary stress (paper speed: 25 mm/s, calibration: 10 mm/mV). (A) EKG obtained approximately one month before admission, showing normal sinus rhythm (heart rate: 93 beats/minute), normal intervals, and a QTc of 422 ms. (B) EKG on admission with sinus tachycardia (heart rate: 104 beats/minute), probable left atrial enlargement, borderline repolarization abnormalities in the lateral leads, and QTc prolongation to 474 ms. These findings reflect evolving cardiac strain in the setting of worsening hypoxia and systemic disease progression.

Electrocardiographic assessment on admission showed sinus tachycardia (heart rate of 104 beats/minute), probable left atrial enlargement, borderline repolarization abnormalities in the lateral leads, and a prolonged QTc (474 ms). Compared to a baseline EKG obtained one month prior (showing normal sinus rhythm and QTc of 422 ms), these findings reflected evolving cardiopulmonary stress in the context of progressive hypoxemia and elevated cardiac biomarkers (Figure 2).

**Figure 3:**
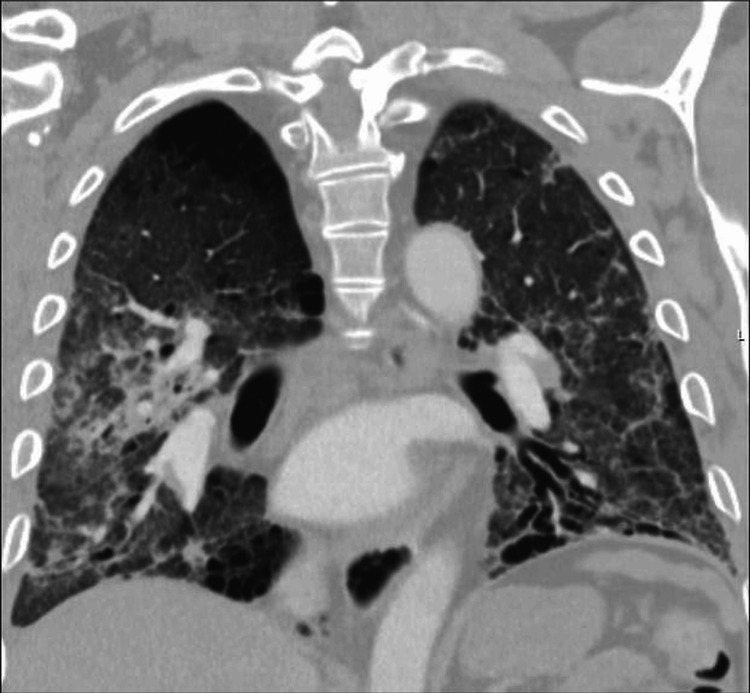
Coronal chest CT. Coronal CT view showing diffuse fibrotic changes with lower lobe predominance, volume loss, and architectural distortion. The main pulmonary artery appears dilated, raising concern for pulmonary hypertension associated with progressive fibrotic interstitial lung disease.

A CT pulmonary angiogram (Figure 3) ruled out pulmonary embolism and demonstrated stable fibrotic changes with interval improvement in ground-glass opacities, residual consolidation in the right upper lobe, and a dilated main pulmonary artery.

**Figure 4:**
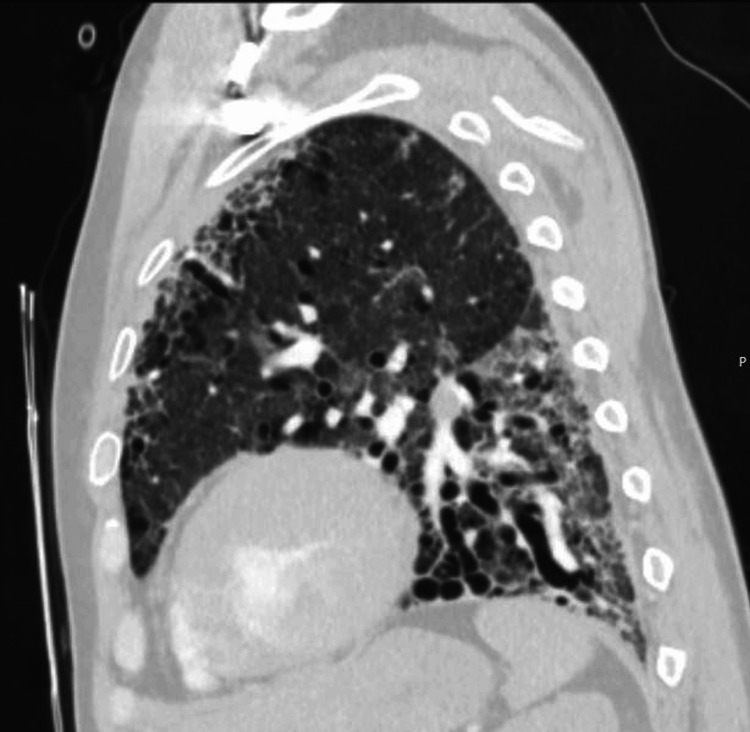
Sagittal chest CT. Sagittal reconstruction highlighting basilar-predominant fibrosis with traction bronchiectasis. No definitive honeycombing is seen. The distribution and imaging features are consistent with a fibrotic non-specific interstitial pneumonia pattern.

These findings were consistent with the progression of underlying fibrotic ILD and possible pulmonary hypertension. Figure 4 further illustrates the basilar predominance of fibrosis and traction bronchiectasis on sagittal views, consistent with fibrotic NSIP without definitive honeycombing.

Serial chest radiographs showed no acute changes. Microbiologic workup, including blood cultures, respiratory viral panel, urine antigens (*Legionella* and *Streptococcus pneumoniae*), and serum β-D-glucan (Fungitell assay), was negative. SARS-CoV-2 PCR was intermittently positive but was interpreted as persistent shedding following recent COVID-19 infection. Autoimmune workup, including prior myositis panel, antinuclear antibody, anti-Scl-70, and anticentromere antibody, remained consistent with known diffuse systemic sclerosis.

Taken together, the diagnostic findings supported a picture of progressive fibrotic ILD rather than an acute infectious process, guiding the team toward escalation of immunosuppressive therapy and lung transplant referral.

Therapeutic intervention

At the time of admission, the patient was maintained on his chronic immunosuppressive regimen of mycophenolate mofetil 1,500 mg twice daily and prednisone 20 mg daily. Given his significant oxygen requirement (up to 15 L/minute) and minimal exertional tolerance, he was managed with high-flow oxygen via a face mask per his preference, chest physiotherapy, bronchodilators, and supportive care. Diuresis was attempted to optimize pulmonary status, and empiric antibiotics were initially deferred due to low clinical suspicion for infection.

Given his ongoing clinical deterioration despite standard therapy and no evidence of an infectious trigger, a multidisciplinary team, including pulmonology, rheumatology, and infectious disease, agreed to escalate immunosuppression. Intravenous cyclophosphamide was initiated at a dose of 750 mg with a plan for monthly infusions for six months, following established protocols for refractory SSc-ILD. Prednisone was cautiously increased to 30 mg daily with close monitoring, acknowledging the heightened risk of scleroderma renal crisis. The patient remained normotensive during admission and continued his baseline antihypertensive regimen (sacubitril-valsartan 24/26 mg twice daily, carvedilol 25 mg twice daily, and nifedipine ER 90 mg daily) for blood pressure control and renal protection. Dapagliflozin (Farxiga) 10 mg daily was also continued for heart failure support, and mycophenolate mofetil was temporarily maintained during the initiation of cyclophosphamide, with plans to taper based on tolerability and response.

Referral for lung transplantation evaluation was initiated; however, the process was delayed when it became clear that the patient’s insurance was not accepted at the local tertiary transplant center. Plans were made to establish care at an alternate center, but the delay underscored a major barrier in securing timely, definitive treatment.

## Discussion

SSc-ILD has become a leading cause of morbidity and mortality among patients with diffuse cutaneous disease, reflecting a shift from earlier decades when renal crisis and pulmonary hypertension were more dominant contributors [1,4]. Although mycophenolate mofetil has emerged as the preferred first-line treatment [5], a subset of patients experience disease progression despite appropriate therapy. This case exemplifies the complexity of managing refractory SSc-ILD and the consequences of delays in definitive care.

The patient’s clinical trajectory, marked by worsening dyspnea, rising oxygen needs, and diminished functional capacity, occurred despite adherence to mycophenolate mofetil and low-dose prednisone. At approximately one month into therapy, he reported persistent dyspnea without evidence of infection, prompting an increase in mycophenolate mofetil to 1,500 mg twice daily. A summary of key clinical events and interventions is presented in Table 2.

**Table 2:** Timeline of clinical course and therapeutic interventions. Clinical events are presented in relative time from the initial diagnosis of systemic sclerosis-associated interstitial lung disease. This timeline illustrates the evolving symptom burden, oxygen needs, and therapeutic adjustments over a 12-month period, highlighting delays in transplant referral and the impact of post-COVID-19 functional decline. SSc-ILD = systemic sclerosis-associated interstitial lung disease; ILD = interstitial lung disease; CAP = community-acquired pneumonia; RHC = right heart catheterization; PH = pulmonary hypertension

Relative time	Event/Context	Therapeutic intervention
Baseline	Diagnosis of diffuse SSc-ILD (NSIP pattern), esophageal dysmotility, Raynaud’s	Prednisone 30 mg daily; mycophenolate mofetil 500 mg BID
+1 month	Symptom flare without infection	Mycophenolate increased to 1,500 mg BID
+2 months	Hospitalization for herpes zoster and ILD exacerbation	Mycophenolate held; methotrexate started; 4 L oxygen
+5 months	Methotrexate discontinued due to affordability	Mycophenolate resumed at 1,500 mg BID
+6 months	Progressive dyspnea after presumed CAP	Home oxygen increased to 5 L/minute
+7 months	CT chest showed fibrotic progression	Continued immunosuppression
+8–9 months	Worsening oxygen requirements	Home oxygen increased to 10 L/minute
+10 months	ED presentation with dyspnea; hospitalized	Rituximab considered; transplant referral initiated
+11 months	Ineligible for transplant at the primary center due to insurance	Referral deferred
+11.5 months	Hospitalized with COVID-19	Treated with remdesivir and baricitinib
+11.6 months	Post-COVID-19 worsening functional status	Home oxygen increased to 15 L/minute
+11.8 months	Readmission for worsening hypoxia	RHC showed precapillary PH; cyclophosphamide started; prednisone tapered
+12 months	Insurance changed; transplant referral resumed	Continued immunosuppressive therapy

Although he was less than a year into immunosuppressive treatment, his rapid decline challenged the conventional expectation that response should be judged after 12 months. In similar cases, where physiologic deterioration outpaces radiologic findings, waiting for treatment milestones can risk missing a narrow window for transplant referral.

His disease course aligned with what has been described as a progressive fibrosing ILD phenotype, characterized by unrelenting respiratory symptoms, functional decline, radiographic progression, and early mortality, regardless of the underlying ILD [6]. Recognizing this phenotype early is essential to the timely escalation of therapy and consideration of lung transplantation, as its clinical behavior often mirrors that of idiopathic pulmonary fibrosis [7].

Importantly, the patient’s deterioration followed a recent SARS-CoV-2 infection, raising the possibility that viral injury accelerated his fibrotic burden. Post-viral fibrotic progression has been increasingly reported in ILD populations, particularly in those with underlying autoimmunity. Yet, despite interval radiographic improvement in ground-glass opacities, his oxygen requirement continued to escalate (from 6-8 L/minute to 15 L/minute at rest), highlighting a disconnect between imaging findings and physiologic deterioration. This clinical-radiographic dissociation reinforced the urgency of transplant evaluation. In fibrosing ILDs, disease progression may be evident through worsening symptoms, functional decline, or increasing oxygen requirements, even in the absence of radiographic change [5].

His increasing dependence on supplemental oxygen, rising from 6-8 L at baseline to 15 L at rest, was among the clearest clinical signals of decline. In the absence of other reversible triggers, it reflected worsening gas exchange and underscored the urgency to act.

Immunosuppressive therapy was escalated to intravenous cyclophosphamide, consistent with recommendations from the European League Against Rheumatism (EULAR) for SSc-ILD refractory to mycophenolate mofetil [8], and in line with more recent consensus updates highlighting its continued role in select patients [5]. The increase in prednisone to 30 mg daily was made judiciously, with close monitoring given his elevated risk for scleroderma renal crisis [9]. This nuanced therapeutic balancing act highlights the value and limits of multidisciplinary input [10]. Pulmonology, rheumatology, cardiology, and infectious disease were all engaged, but even this robust coordination could not overcome a system-level obstacle that proved most consequential.

Despite a clear clinical need, the patient was unable to pursue transplant evaluation at the regional referral center because his insurance was not accepted. The delay that followed is more than an administrative inconvenience; it may have directly threatened his transplant eligibility. For patients with fibrosing ILD, disease progression can be non-linear, and delays in evaluation risk accumulating irreversible complications such as pulmonary hypertension, muscle wasting, or malnutrition, all of which may render a patient ineligible by the time access is secured [11].

Repeated hospitalizations also took a visible psychosocial toll. The patient voiced frustration with both the perceived futility of his hospitalizations and lapses in care delivery. These affective dimensions are more than emotional undercurrents; they can undermine trust in the system and influence adherence and follow-up. His experience serves as a reminder that structural inequities do not merely delay care; they erode the therapeutic relationship [12].

This case invites broader reflection. It challenges us to reconsider how we define treatment failure in ILD, how we integrate transplant referral into chronic disease management, and how we structure our health systems to ensure that advanced therapies are equitably accessible. As clinical tools for fibrosing ILD improve, the systems that deliver them must evolve in parallel.

## Conclusions

This case illustrates the complex intersection of clinical progression and systemic limitation in the care of a patient with diffuse SSc-ILD. Despite adherence to guideline-directed medical therapy and multidisciplinary management, the patient experienced continued clinical deterioration that ultimately required escalation to intravenous cyclophosphamide. The progression occurred in the setting of a recent COVID-19 infection and was marked by a disconnect between radiographic appearance and functional capacity, highlighting the limitations of imaging as a sole marker of disease activity. Crucially, the patient’s inability to access a lung transplant center due to insurance restrictions delayed definitive care in a disease where timing is often critical. This case underscores not only the clinical challenges of managing refractory SSc-ILD but also the systemic inequities that impact treatment access. As clinicians, recognizing and addressing these barriers is essential to providing equitable and timely care for patients with life-limiting conditions.
